# Supplementary material for: Syndrome of Transient Headache and Neurologic Deficits with Cerebrospinal Fluid Lymphocytosis (HaNDL): HHV-7 Finding in Cerebrospinal Fluid Challenges Diagnostic Criteria
Source: Pathogens. 2023 Mar 17;12(3):476. doi: 10.3390/pathogens12030476 (PMC10051435; doi:10.3390/pathogens12030476)
Supplement: Supplementary file 1 [file pathogens-12-00476-s001.zip › Table S1_resubmit.pdf]

Table S1. Laboratory screening tests over time for infectious and autoimmune disorders

Laboratory tests (reference value)	Day 1	Day 4	Day 28	Day 48
**CSF analysis**				
**Bacterial culture**	neg	N/A	neg	N/A
**PCR CMV**	neg	N/A	N/A	N/A
**PCR EBV**	neg	N/A	N/A	N/A
**Enterovirus RNA**	neg	neg	neg	N/A
**PCR HSV 1 and HSV2**	neg	neg	neg	N/A
**PCR VZV**	neg	neg	N/A	N/A
**PCR HHV-6A and HHV-6B**	neg	N/A	neg	N/A
**Borrelia IgM and IgG**	N/A	neg	neg	N/A
**Syphilis TPPA**	N/A	neg	neg	N/A
**Pneumococcus DNA**	N/A	N/A	neg	N/A
**Meningococcus DNA**	N/A	N/A	neg	N/A
**Haemophilus influenzae**	N/A	N/A	neg	N/A
**ACE (<2.0 E/L)**	N/A	N/A	<2.0	N/A
**HHV-7- DNA**	N/A	N/A	pos*	N/A
**Serum analysis**				
**TBE virus IgM and IgG**	neg	N/A	N/A	N/A
**Borrelia IgM**	N/A	neg	Neg	N/A
**Borrelia IgG**	N/A	pos	pos	N/A
**HIV1/HIV2-Ag/Ab**	N/A	neg	N/A	N/A
**Syphilis TPPA**	N/A	N/A	neg	N/A
**Neuronal antibodies**	neg	N/A	N/A	N/A
**Paraneoplastic antibodies**	neg	N/A	N/A	N/A
**ACE (E/L) normal <70**	N/A	18.0	18.3	N/A
**CEA (ug/L) normal <4.7**	N/A	<1.0	N/A	N/A
**Urine analysis**				

**Bacterial culture**	N/A	neg	N/A	N/A
**Chlamydia trachomatis DNA**	N/A	neg	N/A	N/A
**Gonococcus DNA**	N/A	neg	N/A	N/A
**Nasopharynx test**				
**Coronavirus SARS-CoV-2-RNA**	N/A	neg	N/A	N/A

Abbreviations: Ab = antibodies; ACE = angiotensin-converting enzyme; Ag = antigen; CEA = carcinoembryonic antigen; CMV= Cytomegalovirus; CSF = cerebrospinal fluid; DNA = deoxyribonucleic acid; EBV = Epstein-Barr virus; N/A = not available; HHV=human herpes virus; HIV = human immunodeficiency virus; HSV= herpes simplex virus; Ig = immunoglobulin; ug = microgram; ml= milliliter; N/A = Not available; RNA = ribonucleic acid; SARS-CoV-2 = severe acute respiratory syndrome coronavirus 2 (covid-19); TBE = tick-borne encephalitis; TPPA = treponema pallidum particle agglutination assay; u = urine; VZV = varicella-zoster virus

*1.70, 10-log by number of genome equivalents/ml
