# Supplementary material for: Syndrome of Transient Headache and Neurologic Deficits with Cerebrospinal Fluid Lymphocytosis (HaNDL): HHV-7 Finding in Cerebrospinal Fluid Challenges Diagnostic Criteria
Source: Pathogens. 2023 Mar 17;12(3):476. doi: 10.3390/pathogens12030476 (PMC10051435; doi:10.3390/pathogens12030476)
Supplement: Supplementary file 1 [file pathogens-12-00476-s001.zip › Table S2.pdf]

Table S2. Examinations performed

Examination	Finding
CT scan of the brain and angiography (day 1 and day 16)	Normal
Urine toxicology screening (day 1)	Positive for opioids*<br>Positive for THC**
Magnetic tomography inclusive intravenous contrast and spectroscopy (day 3)	Normal
EEG (day 4)	Mild to a moderate focal abnormality with slower wave activity in the left temporal region.
Immunodeficiency panel, B and T cells (day 48)	Normal

Abbreviations: CT = computed tomography, EEG = electro encephalography, THC = (delta-9-tetrahydrocannabinol.

* the patient had received 5 mg morphine and 0.3 mg alfentanil during transportation to the hospital

** the patient had smoked cannabis two days prior to admission.
