# Supplementary material for: Syndrome of Transient Headache and Neurologic Deficits with Cerebrospinal Fluid Lymphocytosis (HaNDL): HHV-7 Finding in Cerebrospinal Fluid Challenges Diagnostic Criteria
Source: Pathogens. 2023 Mar 17;12(3):476. doi: 10.3390/pathogens12030476 (PMC10051435; doi:10.3390/pathogens12030476)
Supplement: Supplementary file 1 [file pathogens-12-00476-s001.zip › Table S3.pdf]

Table S3. HaNDL diagnostic criteria (7.3.5) in ICHD-3 chapter 7.3 Headache attributed to non–infectious inflammatory intracranial disease

ICHD-3 HaNDL criteria	Text of each criterion	Day 1-27	Day 28-48
**A**	Episodes of migraine-like headache fulfilling criteria B and C[1]	Yes	Yes
**B**	Both of the following (B1 and B2)	Yes	Yes
**B1**	accompanied or shortly preceded by onset of at least one of the following transient neurological deficits lasting >4 hours	Yes	Yes
**B1a**	Hemiparaesthesia	Yes	Yes
**B1b**	Dysphasia	Yes	Yes
**B1c**	Hemiparesis	Yes	Yes
**B2**	Associated with cerebrospinal fluid (CSF) lymphocytic pleocytosis (>15 white cells per μl), with negative aetiological studies	Yes	Yes
**C**	Evidence of causation demonstrated by either or both of the following (C1, C2)	Yes	Yes
**C1**	Headache and transient neurological deficits have developed or significantly worsened in temporal relation to onset or worsening of the CSF lymphocytic pleocytosis, or led to its discovery	Yes	Yes
**C2**	Headache and transient neurological deficits have significantly improved in parallel with improvement in the CSF lymphocytic pleocytosis	Yes	Yes
**D**	Not better accounted for by another ICHD-3 diagnosis	No	No
**Fulfillment ICHD-3 HaNDL criteria**		Yes	No*

Abbreviations: ICHD-3, International Classification of Headache Disorders (3rd ed.); HaNDL, syndrome of transient headache and neurologic deficits with cerebrospinal fluid lymphocytosis *Positive aetiological studies:low concentration of HHV-7 in CSF.
