# Supplementary material for: Syndrome of Transient Headache and Neurologic Deficits with Cerebrospinal Fluid Lymphocytosis (HaNDL): HHV-7 Finding in Cerebrospinal Fluid Challenges Diagnostic Criteria
Source: Pathogens. 2023 Mar 17;12(3):476. doi: 10.3390/pathogens12030476 (PMC10051435; doi:10.3390/pathogens12030476)
Supplement: Supplementary file 1 [file pathogens-12-00476-s001.zip › Table S4.pdf]

Table S4. Fulfillment of ICHD-3 diagnostic criteria 9.1.2*Headache attributed to viral meningitis or encephalitis in chapter 9. Headache attributed to infection

**ICHD-3 criteria Headache attributed to viral meningitis or encephalitis**	**Text of each criterion**	**Day 1-27**	**Day 28-48**
**A**	Any headache fulfilling criterion C	No	No
**B**	Viral meningitis or encephalitis has been diagnosed	No	No
**C**	Evidence of causation demonstrated by at least two of the following	No	No
**C1**	headache has developed in temporal relation to the onset of the viral meningitis or encephalitis	No	No
**C2**	headache has significantly worsened in parallel with worsening of the viral meningitis or encephalitis	No	No
**C3**	headache has significantly improved in parallel with improvement in the viral meningitis or encephalitis	No	No
**C4**	headache is either or both of the following: holocranial or located in nuchal area	No	No
**D**	Not better accounted for by another ICHD-3 diagnosis	No	No
**Fulfillment ICHD-3, 9.1.2 criteria**		No	No

Abbreviations: ICHD-3, International Classification of Headache Disorders (3rd ed.); ***Description ICHD-3, 9.1.2 Headache attributed to viral meningitis or encephalitis:** typically with neck stiffness and fever and variably associated, according to the extent of the infection, with neurological symptoms and/or signs including changes in mental state.
