# Supplementary material for: Syndrome of Transient Headache and Neurologic Deficits with Cerebrospinal Fluid Lymphocytosis (HaNDL): HHV-7 Finding in Cerebrospinal Fluid Challenges Diagnostic Criteria
Source: Pathogens. 2023 Mar 17;12(3):476. doi: 10.3390/pathogens12030476 (PMC10051435; doi:10.3390/pathogens12030476)
Supplement: Supplementary file 1 [file pathogens-12-00476-s001.zip › Table S5.pdf]

Table S5. Comparison of virus encephalitis HaNDL symptoms in the current case

**Symptom**	**Viral encephalitis**	**Present HaNDL case**
Neck stiffness	Yes	No
Fever	Yes	No
Neurological symptoms	Yes	Yes
Changes in mental state	Yes	Yes

Abbreviations: HaNDL, Syndrome of transient headache and neurologic deficits with cerebrospinal fluid lymphocytosis.
